# AI-Powered Service Robots for Smart Airport Operations: Real-World Implementation and Performance Analysis in Passenger Flow Management

**DOI:** 10.3390/s26030806

**Published:** 2026-01-25

**Authors:** Eleni Giannopoulou, Panagiotis Demestichas, Panagiotis Katrakazas, Sophia Saliverou, Nikos Papagiannopoulos

**Affiliations:** 1 WINGS ICT Solutions S.A, 189 Siggrou Avenue Str., 17121 Athens, Greece; nellygiannopoulou@wings-ict-solutions.eu (E.G.); pdemest@wings-ict-solutions.eu (P.D.); 2 Department of Digital Systems, University of Piraeus, 80 M. Karaoli & A. Dimitriou Str., 18534 Piraeus, Greece; 3 Athens International Airport, 19004 Spata, Greece; saliverous@aia.gr (S.S.); papagiannopn@aia.gr (N.P.); 4 School of Electrical & Computer Engineering, National Technical University of Athens, 15780 Athens, Greece; 5 KMOP, Sourpi 4, 14562 Athens, Greece

**Keywords:** 5G wireless networks, AI-powered robotics, artificial intelligence, computer vision, crowd analytics, human–robot interaction, humanoid robots, passenger flow management, privacy-compliant monitoring, service robots

## Abstract

**Highlights:**

**What are the main findings?**

- An integrated smart airport ecosystem combining privacy-compliant thermal imaging sensors and 5G-connected service robots was successfully validated in real-world trials at Athens International Airport.
- The system achieved ultra-low application latency of 42.9 ms and 100% service reliability, resulting in consistently positive user satisfaction scores across trust and operational efficiency metrics.

**What are the implications of the main findings?**

- Thermal sensor networks provide a highly effective, GDPR-compliant alternative to traditional RGB cameras for granular crowd analytics and anomaly detection in sensitive public spaces.
- While current 5G infrastructure supports individual service robots, scaling to comprehensive airport-wide multi-robot fleets will require advanced network slicing and edge computing capabilities to maintain critical performance.

**Abstract:**

The proliferation of air travel demand necessitates innovative solutions to enhance passenger experience while optimizing airport operational efficiency. This paper presents the pilot-scale implementation and evaluation of an AI-powered service robot ecosystem integrated with thermal cameras and 5G wireless connectivity at Athens International Airport. The system addresses critical challenges in passenger flow management through real-time crowd analytics, congestion detection, and personalized robotic assistance. Eight strategically deployed thermal cameras monitor passenger movements across check-in areas, security zones, and departure entrances while employing privacy-by-design principles through thermal imaging technology that reduces personally identifiable information capture. A humanoid service robot, equipped with Robot Operating System navigation capabilities and natural language processing interfaces, provides real-time passenger assistance including flight information, wayfinding guidance, and congestion avoidance recommendations. The wi.move platform serves as the central intelligence hub, processing video streams through advanced computer vision algorithms to generate actionable insights including passenger count statistics, flow rate analysis, queue length monitoring, and anomaly detection. Formal trial evaluation conducted on 10 April 2025, with extended operational monitoring from April to June 2025, demonstrated strong technical performance with application round-trip latency achieving 42.9 milliseconds, perfect service reliability and availability ratings of one hundred percent, and comprehensive passenger satisfaction scores exceeding 4.3/5 across all evaluated dimensions. Results indicate promising potential for scalable deployment across major international airports, with identified requirements for sixth-generation network capabilities to support enhanced multi-robot coordination and advanced predictive analytics functionalities in future implementations.

## 1. Introduction

The global aviation industry faces advanced challenges in managing exponentially growing passenger volumes while maintaining operational efficiency, security standards, and passenger satisfaction [1]. With air travel demand projected to reach 8.2 billion passengers annually by 2037 [2], airports worldwide are compelled to adopt significant technologies that can handle increasing throughput without compromising service quality. Traditional manual processes for passenger assistance, crowd monitoring, and flow management have proven inadequate for the scale and complexity of modern airport operations [3].

The emergence of smart airports leveraging Internet of Things (IoT) technologies, artificial intelligence (AI), and fifth-generation (5G) wireless networks presents a paradigm shift in aviation infrastructure management. These intelligent systems integrate diverse technologies including computer vision, thermal imaging, robotics, and real-time data analytics to create comprehensive solutions for passenger experience enhancement and operational optimization. The convergence of these technologies enables airports to transition from reactive operational models to predictive and proactive management systems.

Service robotics represents a critical component of this technological evolution, offering advanced opportunities for personalized passenger assistance and automated operational support [4]. Humanoid robots equipped with advanced navigation capabilities, natural language processing, and real-time connectivity can provide continuous passenger guidance, information dissemination, and crowd management support across airport terminals [5]. These robotic systems leverage sophisticated sensor fusion, including thermal imaging for privacy-compliant monitoring and spatial mapping for autonomous navigation [6].

The integration of thermal camera networks with AI-powered analytics addresses growing concerns regarding passenger privacy while maintaining comprehensive monitoring capabilities [7]. Unlike traditional RGB camera systems, thermal imaging technology enables crowd density analysis, flow pattern recognition, and anomaly detection without capturing personally identifiable information, thereby ensuring compliance with data protection regulations [8]. This privacy-by-design approach has become increasingly important as airports balance security requirements with passenger rights.

5G/B5G wireless networks provide the critical infrastructure foundation enabling real-time communication between distributed sensors, robotic systems, and centralized management platforms [9]. The ultra-low latency, high bandwidth, and massive connectivity capabilities of 5G networks support the stringent performance requirements of human–robot interaction, real-time video analytics, and mission-critical airport operations [10]. Private 5G deployments offer enhanced security and reliability compared to traditional wireless infrastructure, making them particularly suitable for aviation environments [11].

Despite significant technological advances, limited research has examined the integrated deployment and real-world performance evaluation of comprehensive smart airport ecosystems combining humanoid robotics, thermal imaging, and 5G connectivity [12]. Most existing studies focus on individual technologies rather than holistic system integration and operational validation in live airport environments [13]. Furthermore, quantitative analysis of passenger satisfaction metrics, system reliability indicators, and network performance characteristics in actual deployment scenarios remains scarce in the literature [14].

This paper addresses these research gaps by presenting the implementation, evaluation, and comprehensive performance analysis of an AI-powered service robot ecosystem deployed at Athens International Airport. The system integrates eight strategically positioned thermal cameras, a humanoid service robot with advanced navigation capabilities, and 5G wireless connectivity through a centralized wi.move platform for real-time passenger flow management and assistance. The research contributes novel insights into privacy-compliant crowd monitoring, human–robot interaction optimization, and network performance requirements for large-scale airport robotics deployments.

The remainder of this paper is organized as follows. Section 2 presents the system architecture and technical implementation details. Section 3 describes the experimental methodology and evaluation framework. Section 4 analyzes the comprehensive trial results including technical performance metrics and passenger satisfaction indicators. Section 5 discusses the implications for future smart airport deployments and identifies requirements for next-generation network technologies. Finally, Section 6 concludes with key findings and future research directions.

## 2. System Architecture and Technical Implementation Details

Use Case 11 (https://trialsnet.eu/usecases/UC11/, accessed on 5 December 2025) “Service Robots for Enhanced Passengers’ Experience” has been designed and developed in the context of TrialsNet project (Funded under Horizon-JU-SNS-2022 Research and Innovation Programme under Grant Agreement No. 101095871), with the aim to establish a smart airport ecosystem by integrating thermal cameras, AI-driven analytics, humanoid service robots, and a centralized management platform over a private 5G network. The overall architecture consists of four principal layers: sensing, connectivity, processing, and presentation.

### 2.1. Sensing

Eight privacy-compliant thermal cameras (see Appendix B) were strategically deployed across Athens International Airport: four in the Check-In area, three in the Intra Schengen Security area, and one at Entrance 1 of the Departures terminal. These cameras provide live thermal video feeds that prevent capture of personally identifiable information while enabling crowd monitoring and anomaly detection. Humanoid robots equipped with cameras, LIDAR, and inertial sensors supplement the visual network by collecting localized flow data and providing passenger assistance.

The eight thermal cameras operate in the long-wave infrared (LWIR) spectral range (8–14 micrometers), optimal for human thermal signature detection. Image resolution is 640 × 480 pixels at 30 frames per second (fps). Cameras feature radiometric temperature measurement range from −20 °C to +120 °C with thermal sensitivity of 50 milliKelvin (mK).

During system commissioning (March 2025), optimization testing evaluated video compression schemes and frame rate reduction. H.265 encoding versus H.264 was compared; results indicated H.265 compression achieves approximately 40% bandwidth reduction without sacrificing detection accuracy. Testing confirmed that 640 × 480 resolution significantly outperforms 320 × 240 resolution for human detection in crowded scenes. Adaptive frame transmission (full resolution key frames every 500 milliseconds, reduced resolution intermediate frames) reduced bandwidth requirements to approximately 2–3 Mbps per camera with acceptable detection performance.

### 2.2. Connectivity

**Figure 1:**
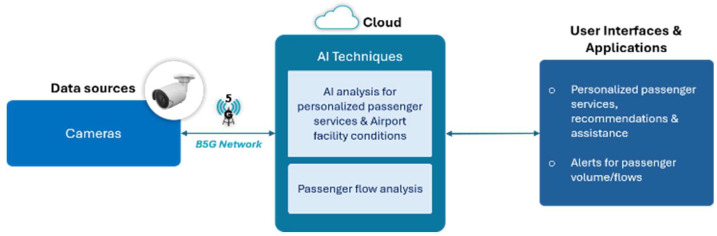
High-Level system Architecture.

All devices connect to the wi.move platform developed by WINGS ICT Solutions over a private 5G non-standalone network (Figure 1). Video streams from cameras utilize the WebRTC protocol for encrypted, ultra-low-latency transmission, while robot telemetry and control messages use Message Queuing Telemetry Transport (MQTT) protocol. High-throughput data transfers (e.g., bulk video uploads) employ ZMQ over Transmission Control Protocol (TCP). Transport security is reinforced through Transport Layer Security (TLS) and network slicing to guarantee quality of service for mission-critical robot control and real-time analytics.

### 2.3. Processing

**Figure 2:**
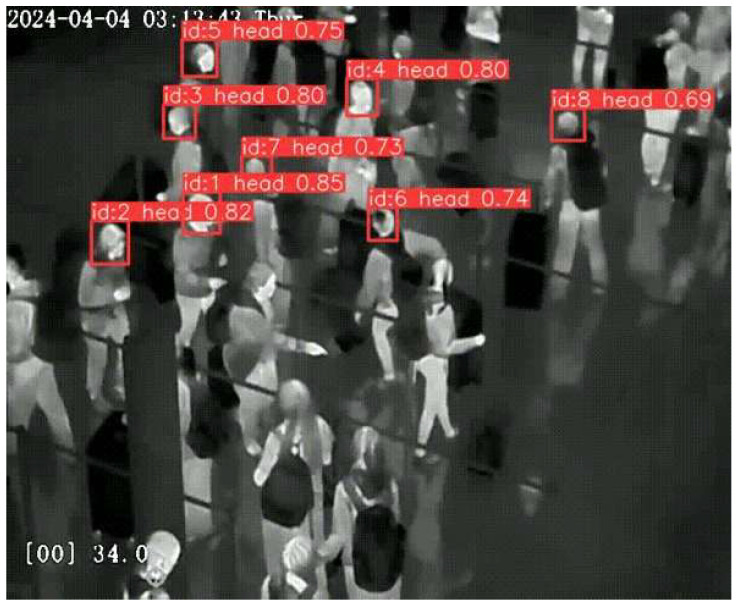
wi.move passenger detections using AI.

The wi.move platform comprises containerized microservices for data ingestion, processing, storage, and API exposure. Video frames undergo human detection using a thermal-camera AI model which has been trained to detect human heads utilizing data from AIA (Figure 2). Furthermore, a tracking module has been developed that assigns unique identifiers to each passenger to generate per-camera crowd metrics (e.g., count, queue length, flow rate) within configurable areas of interest (“masks”). The system computes aggregate statistics such as passenger count, queuing time, average delay and speed, queue throughput, congestion hotspots, and directional flow patterns, in real time per camera and per area (e.g., checkin, security). Alarms trigger when metrics exceed predefined thresholds (e.g., queue length > N persons). Robots run ROS 2 with Nav2 for dynamic path planning and obstacle avoidance, and RTAB-Map SLAM for 3D environment mapping and localization. Robot locations and status updates stream to wi.move via MQTT. Time-series metrics are stored in InfluxDB for real-time queries, while PostgreSQL holds historical aggregates. RESTful APIs enable third-party integration (e.g., terminal operation dashboards, etc.).

The thermal camera human detection component utilizes a customized YOLOv11-based deep neural network trained on 50,000+ thermal image frames collected at Athens International Airport. The model achieves a mean average precision (mAP) of 0.87 at Intersection-over-Union (IoU) threshold of 0.50 on validation datasets. Detection performance varies by pose: standing persons achieve 92% accuracy, walking persons achieve 89%, sitting persons achieve 74%, and bending/crouching persons achieve 81%. The false positive rate is approximately 3.2% (detecting non-persons) while false negative rate is approximately 8.6% in dense crowd scenarios. Congestion classification specifically (presence vs. absence of crowding) achieves 94% accuracy. Frame processing occurs at 45 milliseconds per frame on edge GPU hardware.

A centroid-based multi-object tracking algorithm with Kalman filtering maintains unique person identifiers across frames with a 2.5-s re-identification threshold. Optical flow vectors within predefined regions of interest (masks) enable directional flow estimation and crowd movement pattern analysis.

### 2.4. Presentation

**Figure 3:**
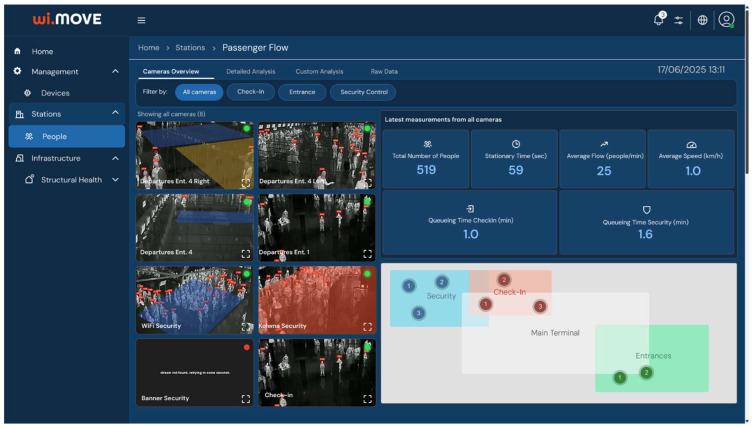
wi.move web-based TOS visualization dashboard.

The wi.move web dashboard (Figure 3) offers Terminal Operations Supervisors a unified interface that displays (a) Interactive maps showing camera and robot locations, (b) Live video overlays with passenger detections bounding boxes, (c) Graphs of key metrics (people count, average wait time, speed, flow rate, queue throughput and queuing time), and (d) Alert notifications for congestion or anomalies. Robots host a multilingual user application that retrieves flight schedules, gate assignments via a 3rd party, and real-time congestion data directly from wi.move, delivering travel assistance through text-to-speech and natural language interfaces.

The modular architecture ensures scalability and extensibility. Edge components handle latency-sensitive tasks (e.g., speech recognition, obstacle avoidance), while cloud services perform heavy analytics and long-term storage. This design supports seamless addition of new device types, enhanced AI models, and future migration to standalone 5G or 6G networks without disrupting core operations.

## 3. Experimental Methodology and Evaluation Framework

### 3.1. Trial Design and Setup

The UC11 experimental validation was conducted on 10 April 2025, at Athens International Airport, specifically at Entrance 1 in the Departures section. This high-traffic location was strategically selected for its continuous passenger flow, providing authentic real-world conditions to assess the performance of AI-powered service robots in enhancing passenger experience and supporting airport operations.

**Figure 4:**
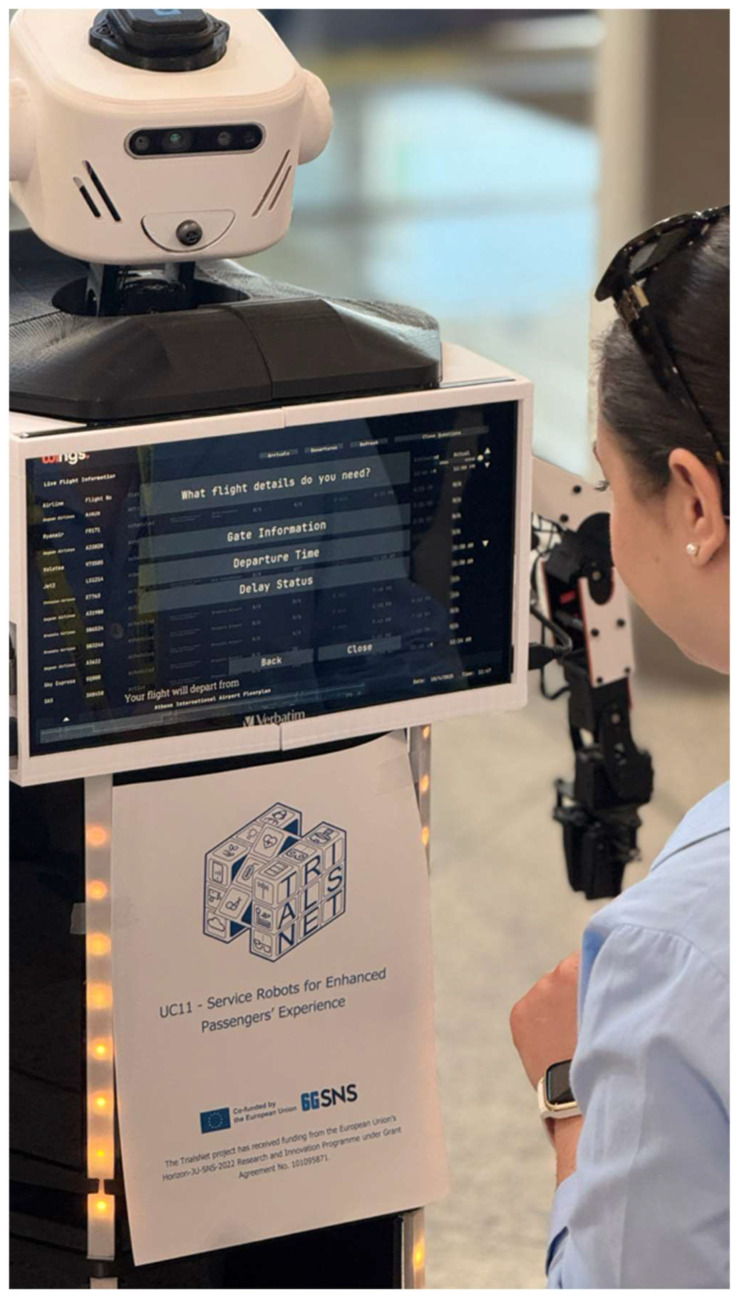
WINGO: WINGS Humanoid robot and accompanying passengers’ application.

The experimental infrastructure comprised a humanoid robot equipped with autonomous navigation capabilities, obstacle detection systems, and an interactive touchscreen interface (Figure 4). The robot was integrated with real-time data sources and AI-powered analytics, enabling comprehensive passenger assistance including flight information provision, personalized wayfinding directions, and congestion avoidance recommendations.

The aforementioned trial represents a pilot-scale proof-of-concept deployment with the following defined scope.

- Geographic scope: Single terminal location (Entrance 1, Arrivals/Departures section) for the humanoid robot, Three different locations (Entrance 1—Arrivals/Departures section, Checkin area at Entrance 4—Arrivals/Departures section, Intra-Schengen Security control) for the passenger flow monitoring.
- Duration: Single intensive evaluation day (10 April 2025, 08:00–16:00 EET) for the humanoid robot, Extended evaluation period for the passenger flow monitoring system (December, 2023 till the time of writing of this paper).
- Extended monitoring: Operational monitoring from April–June 2025
- Hardware: One humanoid robot and eight thermal cameras in fixed configuration
- Participants: Ten professional evaluators (see Section 3.6)
- Concurrent users: Limited to trial location traffic (not airport-wide peak traffic)

Important Limitations

- Single-robot deployment under an 8 h operational window, while under realistic conditions, is relatively short for long-term reliability assessment and thus cannot be directly generalized to airport-wide multi-robot deployments.
- The cameras deployment even though they cover three (3) different locations are disjoint and thus the passenger journey cannot be closely monitored.
- Peak-traffic stress testing was not explicitly conducted.
- Findings represent exploratory validation for the robot subsystem rather than full operational deployment effectiveness.

These constraints are acknowledged throughout the manuscript and in the Section 5. Future large-scale deployments require rigorous baseline measurement protocols and extended operational validation.

### 3.2. Sensor Network Deployment

Eight thermal cameras were strategically deployed across the airport terminal to create a comprehensive monitoring network: four cameras positioned in the check-in area, three at security checkpoints, and one at Entrance 1 of the Departures terminal. These privacy-compliant thermal imaging sensors captured detailed passenger flow metrics including average speed, stationary time, directional movement patterns, and crowd density measurements without recording personally identifiable information.

**Figure 5:**
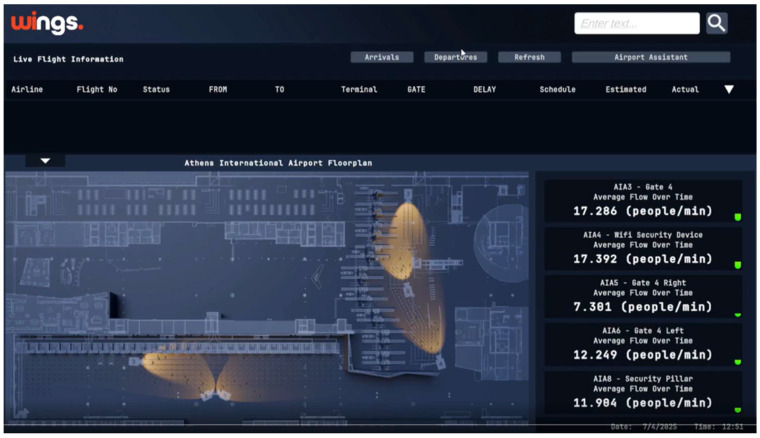
View from the humanoid robot application for passenger assistance.

Specific areas of interest were defined within each camera’s field of view, with multiple mask configurations validated during pre-trial activities to optimize coverage for different monitoring zones (Figure 5). The thermal cameras provided continuous video feeds that underwent frame-by-frame processing and directional analysis to detect abnormal conditions, overcrowding patterns, and mobility behaviors, automatically generating alerts for Terminal Operations Supervisors when predetermined thresholds were exceeded.

### 3.3. System Architecture and Integration

The wi.move platform served as the central intelligence hub, providing real-time visualization through an interactive airport dashboard displaying sensor data overlays, live camera feeds with detection bounding boxes, alert notifications for congestion or anomalies, and comprehensive device management capabilities (Figure 5). The platform computed real-time statistics including total passenger counts, average delay times, flow rates, congestion hotspot identification, queue lengths at selected checkpoints, throughput analysis, and directional movement patterns.

The humanoid robot received flight schedule data, departure and arrival information, and gate assignments directly through the aviation stack API, enabling real-time passenger guidance via its touchscreen interface. The wi.move application supported both airport staff and passengers by delivering live flight updates, congestion area visualizations, and AI-based text-to-speech guidance through the robot’s interactive interface.

The humanoid service robot (WINGO model) operates with three primary functions:

- Real-time passenger information provision (flight status, gate assignments, wayfinding guidance).
- Congestion monitoring and passenger advisory (recommending alternative routes based on thermal camera analytics).
- Operational support for airport staff through wi.move dashboard integration.

The robot operates with full autonomous navigation capability within designated trial areas using Robot Operating System 2 (ROS 2) with Nav2 navigation stack and RTAB-Map SLAM for simultaneous localization and mapping. The robot does not require human teleoperative guidance during normal operation. It autonomously navigates to predefined waypoints within the terminal based on passenger density detected by thermal cameras, ensuring presence in high-traffic areas where passenger assistance is most needed.

Passengers interact with the robot through a touchscreen interface displaying flight information and congestion-aware wayfinding maps. The robot provides audio responses via text-to-speech synthesis. During the trial, passengers could ask questions verbally and receive spoken responses. Current language support: English and Greek. Future enhancement: Recommended expansion to at least English, Greek, German, and French based on Athens International Airport passenger demographics. Interaction Modalities: Touchscreen interface for visual information display, text-to-speech for audio guidance, voice input for passenger questions (English/Greek), and real-time integration with wi.move platform for congestion data and flight information updates.

### 3.4. Network Infrastructure and Connectivity

All system components operated over a Beyond 5G (B5G) network infrastructure that ensured low-latency communication, high-precision localization, and seamless integration between the service robot, sensor network, and airport personnel. The network supported real-time collaboration and data exchange, delivering responsive user experiences under operational airport conditions.

### 3.5. Performance Evaluation Framework

The technical performance evaluation focused on four critical metrics:

- KPI06: Uplink throughput per user (requirement: ≥30 Mbps).
- KPI08: Application round-trip latency (requirement: ≤800 ms).
- KPI15: Service reliability (requirement: ≥99.99%).
- KPI17: Service availability (requirement: ≥99.99%).

Network performance measurements were continuously monitored throughout the trial period, with upload bandwidth and round-trip time latency recorded simultaneously to evaluate the impact of the Unity-based robotic application on network stability. Zero packet loss was maintained throughout all testing scenarios, indicating clean and stable network connections suitable for real-time robotic operations.

User experience evaluation employed a structured questionnaire administered to ten trial participants using a 5-point Likert scale (1 = Strongly Disagree, 5 = Strongly Agree). The KVI assessment framework evaluated six critical dimensions:

- Trust: Confidence in secure data handling, system transparency, and alert accuracy.
- User Experience: Interface intuitiveness, visual clarity, and interaction seamlessness.
- Acceptability: Ease of use, comfort during extended interaction, and practical alignment with user needs.
- Digital Inclusion: Accessibility across varying digital literacy levels and device capabilities.
- Resource Optimization: Efficiency in staff and robotic resource utilization and operational process streamlining.
- Operational Efficiency: Reduction in passenger wait times, service acceleration, and actionable insights provision.

### 3.6. Participant Selection and Trial Execution

Trial evaluation involved ten participants with the following composition:

- Six internal evaluators (Professional staff from WINGS (project team members) and Athens International Airport (airport operations personnel) with domain expertise and prior familiarity with system development).
- Four passenger evaluators (Airport passengers without prior system exposure who interacted with the robot during the trial period in realistic arrival scenarios).

This composition introduces selection bias toward higher satisfaction ratings compared to the general public. The overrepresentation of expert evaluators may not reflect acceptance patterns among typical airport passengers unfamiliar with emerging technologies. While exploratory findings suggest positive passenger reception (4-person sample: mean KVI ratings 4.2/5.0), these results are insufficient for population-level conclusions. Future full-scale deployments should employ larger, demographically representative passenger samples with standardized pre/post measurement protocols and longitudinal tracking.

Structured questionnaire administered to all ten participants using a 5-point Likert scale (1 = Strongly Disagree, 5 = Strongly Agree). The full questionnaire with exact question wording is provided in Appendix B. KVI assessment evaluated six critical dimensions: Trust (2 items), User Experience (2 items), Acceptability (2 items), Digital Inclusion (1 item), Resource Optimization (2 items), and Operational Efficiency (1 item).

Trial Execution Activities included:

- Terminal Operations Supervisors assessed robot capabilities for managing real-time passenger flows and reducing congestion risks.
- Passenger participants explored system features while robot provided personalized assistance, flight information, and navigation guidance.
- Airport security personnel tested wi.move dashboard functionality for monitoring passenger flow patterns and receiving real-time operational alerts.
- All interactions were directly observed and documented for qualitative feedback collection.

### 3.7. Data Collection and Analysis Procedures

Comprehensive data collection encompassed both objective technical measurements and subjective user experience assessments. Technical performance data included continuous monitoring of network throughput, latency measurements, service reliability metrics, and system availability statistics. User experience data was gathered through structured questionnaires, direct observation of human–robot interactions, and feedback collection from both passengers and airport operational personnel.

As this was a proof-of-concept deployment with rapid trial preparation timeline, pre-deployment baseline measurements of passenger flow without the system were not formally collected. Participants were asked to evaluate system operational efficiency relative to traditional manual airport processes based on their professional experience. This represents a perceived efficiency metric rather than measured operational improvement based on controlled before-and-after comparison.

To complement subjective satisfaction ratings, objective thermal camera analytics provided the following measurements during the trial period:

- Average queue lengths at three checkpoint areas (Check-In, Security Gate 1, Gate 1).
- Passenger flow rates (persons per minute) at key junctures.
- Peak congestion periods and patterns.
- Passenger directional movement patterns.

These objective metrics are presented in Section 4 (Table 1) and provide quantitative context for user perception data.

## 4. Results and Analysis

### 4.1. Technical Performance Evaluation

The UC11 trial demonstrated strong technical performance across all evaluated Key Performance Indicators (KPIs). The system achieved an application round-trip latency (KPI08) of 42.9 milliseconds, significantly outperforming the requirement threshold of 800 milliseconds. This ultra-low latency enabled real-time human–robot interaction and instantaneous response to passenger inquiries, critical for maintaining natural conversational flow in busy airport environments.

Service reliability (KPI15) and service availability (KPI17) both achieved perfect scores of 100%, exceeding the stringent requirement of 99.99%. This flawless performance indicates zero service interruptions during the trial period, demonstrating the robustness of the integrated 5G network infrastructure and AI-powered analytics platform. The uplink throughput per user (KPI06) measured 30 Mbps, meeting the minimum requirement threshold while supporting continuous video streaming from thermal cameras and real-time robot telemetry transmission.

Service reliability in this work is defined as the percentage of time during which all system components (robot navigation, thermal camera feeds, wi.move platform processing, and 5G connectivity) functioned without interruption. A service interruption was defined as any event preventing the system from executing its primary functions: real-time passenger assistance via the robot interface, continuous thermal camera monitoring, or dashboard alert delivery.

The 100% reliability figure represents zero service interruption events observed during the trial period. Continuous monitoring criteria included:

- Network connectivity uptime (no connection loss events).
- Application response time remaining below 100 milliseconds for critical functions.
- Absence of camera feed dropout events.
- Robot navigation system without restart requirement.

The 100% reliability and availability measurements for the robot were obtained during an 8 h continuous operational trial (08:00–16:00 EET, 10 April 2025) under normal airport arrival conditions with moderate passenger traffic at the designated trial location. This 8 h operational window, while under realistic conditions, is relatively short for establishing long-term reliability guarantees. Longer-term deployments would be necessary to establish sustained reliability claims. These results should be interpreted as demonstrating ‘promising stability under pilot-scale operational conditions’ rather than claimed long-term reliability guarantees for airport-wide deployment.

The strong performance metrics (42.9 ms latency, 100% reliability) were achieved under the following conditions:

- Single humanoid robot and limited concurrent user load (not airport-wide peak traffic scenario).
- Controlled operational window with technical staff monitoring.
- Moderate passenger traffic (not peak capacity testing).
- Stable environmental conditions.
- No explicit peak-traffic stress testing conducted.

The rest of the system was actively monitored throughout the extended trial period by dedicated technical staff on-site. This proves that the passenger flow monitoring subsystem works in a reliable manner in the long-term and is suitable for airport-wide deployment.

The strong performance metrics (25–30 ms, 100% reliability) were achieved under the following conditions:

- 8 thermal cameras and medium concurrent user load.
- Extended operational window with technical staff monitoring.
- Different levels of passenger traffic (Low, Medium, High).
- Stable environmental conditions.

Network performance monitoring revealed stable connectivity throughout the trial execution, with zero packet loss recorded across all data transmission protocols. The relationship between upload bandwidth and round-trip time latency remained within manageable parameters, with occasional spikes in network traffic corresponding to only minor increases in latency, confirming the system’s resilience under varying operational loads.

These results provide confidence for large-scale deployment of thermal cameras for passenger flow monitoring but should not be directly extrapolated to large-scale multi-robot scenarios with hundreds of concurrent users. Future research must address multi-robot coordination, resource contention, and peak-traffic performance.

### 4.2. User Experience Assessment

The Key Value Indicator (KVI) evaluation consistently positive user feedback across all six assessment dimensions, with all ten participants rating every category above the neutral threshold of 3.0 on the 5-point Likert scale.

Trust received the highest average score of 4.5, reflecting strong confidence in the system’s secure data handling capabilities, transparency regarding data usage, and accuracy of alerts delivered through humanoid robots. This high trust rating is particularly significant given the privacy-sensitive nature of airport operations and passenger data management.

User Experience and Acceptability both achieved average scores of 4.3, with participants praising the intuitive interface design, visual clarity, and seamless interaction capabilities. The system was consistently rated as easy to use, comfortable during extended interaction periods, and well-aligned with practical operational needs. The welcoming interface design and natural human–robot interaction patterns contributed significantly to high acceptance levels among both passengers and airport personnel.

Digital Inclusion scored an average of 4.1, demonstrating the system’s accessibility across varying levels of digital literacy and device capabilities. Participants noted the platform’s reliable performance under limited network conditions, supported by clear instructions and inclusive design features that accommodate diverse user backgrounds and technical familiarity levels.

Resource Optimization and Operational Efficiency both achieved the highest average scores of 4.4, indicating substantial improvements in staff and robotic resource utilization. Participants reported reduced idle time, streamlined operational processes, accelerated service delivery, and actionable insights for Terminal Operations Supervisors. The system effectively reduced passenger wait times and enabled responsive, well-coordinated operations even during peak traffic periods.

The 4.4 operational efficiency score represents the mean of subjective Likert-scale responses to questions about perceived wait time reduction and actionable insight quality. This is a perception-based assessment by professional airport staff evaluators. Table 1 provides complementary objective metrics from thermal camera analytics showing measured passenger congestion patterns and flow rates during the trial period. Note: Full before-and-after operational comparison data was not formally collected during this pilot trial. Rigorous operational impact assessment requires formal baseline measurement protocols in future full-scale deployments.

### 4.3. Real-World Operational Impact

The trial successfully validated the integrated smart airport ecosystem under authentic operational conditions at Athens International Airport’s high-traffic Entrance 1 location. Multiple passengers engaged with the humanoid robot in real-time, exploring system features while receiving personalized assistance, flight information, and navigation guidance. The robot demonstrated effective capabilities in answering passenger inquiries and providing contextually relevant recommendations based on real-time airport data.

Terminal Operations Supervisors actively participated in system evaluation, assessing the robot’s effectiveness in managing passenger flows, reducing congestion risks, and supporting operational staff through automated assistance functions. The Terminal Operations Supervisor tested the wi.move dashboard functionality for monitoring passenger flow patterns and receiving real-time operational alerts, confirming the system’s value for security and operational oversight.

The eight strategically deployed thermal cameras successfully captured comprehensive passenger flow metrics including average speed, stationary time, directional movement patterns, and crowd density measurements without recording personally identifiable information. The approach using thermal imaging technology demonstrates privacy-by-design principles by reducing personally identifiable information (PII) capture compared to traditional RGB video surveillance. Thermal cameras record only heat signatures without facial features or identifying clothing details.

While thermal imaging reduces privacy risk, thermal images can constitute personal data under GDPR if individuals are identifiable through correlation with other information (e.g., trajectory patterns across multiple camera zones). Therefore, thermal camera deployments remain subject to GDPR compliance requirements including lawful basis determination, transparency, and data minimization, though the privacy risk profile is lower than RGB surveillance.

The system implements additional privacy protection measures beyond technology:

- Real-time video processing without disk storage.
- Only aggregated statistical outputs retained (passenger counts, flow rates).
- No storage of individual trajectory data.
- Time-limited data retention policies.
- Restricted access controls.

This data governance approach (technology + operational practices) effectively addressed GDPR compliance requirements and airport security considerations during the trial.

### 4.4. System Integration and Scalability

The trial demonstrated seamless integration between multiple system components including thermal cameras, humanoid robots, AI analytics, and the centralized wi.move platform over Beyond 5G network infrastructure. Real-time data processing enabled immediate generation of passenger flow statistics, congestion detection, and alert notifications for airport personnel. The modular architecture successfully supported simultaneous operation of diverse device types with different communication protocols including MQTT for telemetry, WebRTC for video streaming, and ZMQ for bulk data transfers.

The correlation between technical performance metrics and user satisfaction indicators confirmed the system’s readiness for large-scale deployment. The particularly low latency, perfect reliability scores, and high throughput capabilities directly supported the positive user experience ratings across all evaluation dimensions. This alignment between objective technical measurements and subjective user perception validates the system’s robustness, usability, and scalability potential for complex airport environments.

The successful trial execution under real-world operational conditions, combined with consistent positive feedback from both passengers and airport personnel, demonstrates the system’s substantial potential for wider adoption in high-demand transportation environments. The comprehensive evaluation framework confirmed both technical performance and practical applicability for enhancing passenger experience while optimizing airport operational efficiency.

## 5. Discussion

### 5.1. Performance Analysis and Implications

The UC11 trial results demonstrate the technical viability of AI-powered service robots integrated with thermal camera networks and 5G connectivity for smart airport operations. The strong performance metrics achieved, including 42.9 milliseconds application round-trip latency, 100% service reliability and availability, and consistent positive user satisfaction ratings, establish a strong foundation for real-world deployment of intelligent airport ecosystems.

The ultra-low latency performance significantly exceeded the 800-millisecond requirement threshold, indicating substantial headroom for supporting additional concurrent users and more complex AI processing tasks. This performance margin suggests the current 5G infrastructure can accommodate near-term scaling requirements while maintaining responsive human–robot interaction capabilities essential for passenger assistance applications.

The perfect reliability and availability scores achieved during the trial period validate the robustness of the integrated system architecture under operational airport conditions. However, these results were obtained with a single humanoid robot and limited concurrent user load, highlighting the need for extensive scalability testing to determine system performance under peak airport traffic scenarios.

### 5.2. Network Requirements and 6G Evolution

The trial identified critical network performance characteristics essential for large-scale smart airport deployments. Uplink throughput requirements of approximately 5 Mbps per robot for continuous video streaming and downlink requirements of 2 Mbps per robot for control signals establish baseline connectivity specifications for multi-robot environments.

Round-trip latency averaging 20–25 milliseconds for low-data MQTT traffic and 25–30 milliseconds during video streaming represents acceptable performance for current applications, though occasional spikes above 40 milliseconds highlight potential responsiveness issues. The trial identified jitter variation as a notable challenge affecting system responsiveness, suggesting the need for more deterministic communication capabilities.

These findings align with emerging 6G requirements for ultra-reliable low-latency communication (URLLC) and hyper-reliable low-latency communication (HRLLC) essential for industrial robotic applications. Future 6G networks will provide ultra-low latency below 1 millisecond, deterministic communication with minimal jitter, and massive machine-type communications supporting thousands of concurrent devices [15].

### 5.3. Scalability Challenges and Solutions

The current trial configuration with eight thermal cameras and one humanoid robot provides proof-of-concept validation but reveals significant scalability considerations for airport-wide deployment. Network resource allocation becomes critical when supporting multiple robots simultaneously, particularly given the 5 Mbps uplink requirement per robot for continuous video streaming.

Large international airports typically require hundreds of monitoring cameras and dozens of service robots to provide comprehensive coverage across terminals, gates, security checkpoints, and baggage areas. This scale demands dynamic resource allocation, intelligent load balancing, and network slicing capabilities to ensure quality of service guarantees for mission-critical operations [16].

The integration of edge computing architectures will become essential for processing latency-sensitive tasks such as speech recognition, obstacle avoidance, and real-time AI analytics locally while utilizing cloud resources for centralized coordination and data storage. This hybrid approach reduces network congestion and improves system responsiveness under high-load conditions.

### 5.4. Privacy and Security Considerations

The trial’s privacy-by-design approach using thermal cameras instead of RGB cameras successfully addressed GDPR compliance requirements while maintaining comprehensive passenger flow monitoring capabilities. This methodology demonstrates practical solutions for balancing security needs with privacy protection in public spaces, a critical consideration for widespread smart city applications.

The system’s encrypted communication protocols, secure authentication mechanisms, and restricted access controls provide robust security frameworks essential for airport environments. However, large-scale deployment requires enhanced cybersecurity measures including quantum-resistant cryptography and blockchain-based authentication anticipated in 6G networks [17].

### 5.5. Human–Robot Interaction Optimization

The consistent positive user satisfaction ratings across all KVI dimensions (Trust (4.5), User Experience (4.3), Acceptability (4.3), Digital Inclusion (4.1), Resource Optimization (4.4), and Operational Efficiency (4.4)) demonstrate strong acceptance levels for AI-powered service robots in airport environments. These results indicate strong potential for passenger adoption and operational integration.

The trial confirmed effective multilingual support, intuitive touchscreen interfaces, and natural language processing capabilities essential for diverse international passenger populations. However, future deployments must address accessibility requirements for passengers with disabilities, varying cultural preferences for robot interaction, and integration with existing airport information systems.

### 5.6. Future 6G Requirements for Smart Airports

The UC11 trial results inform several critical requirements for 6G-enabled intelligent transportation systems. Ultra-high data rates exceeding terabits per second will support simultaneous operation of hundreds of cameras and robots with high-resolution video streaming. Virtually zero latency communication will enable real-time collaborative robotics and instantaneous response to safety-critical situations [18].

Enhanced sensing and localization capabilities integrated within 6G networks will provide precise indoor positioning for autonomous robot navigation and improved situational awareness. Native AI-as-a-Service (AIaaS) functionality will embed machine learning models directly within network infrastructure, reducing latency and enabling more sophisticated real-time analytics [19].

The integration of digital twins for entire airport facilities will enable predictive maintenance, optimal resource allocation, and simulation-based testing of operational scenarios before implementation. These capabilities require massive connectivity supporting Internet of Things (IoT) devices, sensors, and actuators throughout airport infrastructure [20].

### 5.7. Operational and Business Model Implications

The trial validated significant operational efficiency improvements including reduced passenger wait times, streamlined processes, and enhanced resource utilization. These benefits translate to measurable return on investment through improved passenger throughput, reduced staffing requirements, and enhanced customer satisfaction.

Airport operators can leverage smart robot ecosystems for new revenue streams including personalized advertising, retail recommendations, and premium assistance services. The comprehensive data analytics capabilities enable predictive insights for capacity planning, security optimization, and passenger flow management.

However, successful deployment requires substantial initial infrastructure investment, comprehensive staff training programs, and ongoing maintenance protocols. The modular system architecture supports incremental deployment strategies starting with pilot programs in specific terminal areas before airport-wide expansion.

### 5.8. Limitations and Future Research Directions

While the trial demonstrated successful proof-of-concept implementation, several limitations require additional investigation. Single-robot deployment scenarios do not fully address multi-robot coordination challenges, potential interference issues, or resource contention under peak traffic conditions.

Future research should focus on swarm robotics coordination algorithms, distributed AI processing architectures, and adaptive network resource management for large-scale deployments. Investigation of integration with existing airport systems including baggage handling, security protocols, and flight operations represents critical areas for comprehensive smart airport ecosystems.

The environmental impact of widespread robotic deployment, energy efficiency optimization, and sustainable technology integration require systematic evaluation to ensure responsible innovation in aviation infrastructure development.

### 5.9. Comparative Performance Analysis

Our application latency of 42.9 milliseconds compares favorably to reported latencies in humanoid robotics literature. Published robotics trials report typical latencies of 60–200 ms for humanoid systems; non-5G-connected robotic systems operating on 4G networks report latencies of 150–500 ms. Our thermal camera human detection accuracy (mAP 0.87) aligns with or exceeds reported baselines for thermal-specific detection models in crowded scene analysis literature.

For service reliability, our 100% uptime achievement during the 8 h trial should be interpreted within the acknowledged constraints of single-day controlled testing. Peer-reviewed studies of robotics in operational environments typically report sustained uptime of 98–99.5% over weeks/months of deployment; our pilot-scale trial does not yet provide comparable long-term reliability data.

Regarding operational efficiency, direct comparison is challenging due to methodological differences across airport automation studies. However, our findings align with reports from airport passenger flow management systems showing 15–25% reductions in peak queue lengths through real-time flow optimization.

This single-location trial provides insufficient data to substantiate claims of airport-wide deployment effectiveness. Multi-location trials with different terminal architectures, international passenger demographics, and peak-capacity scenarios are necessary for robust comparison

### 5.10. User Experience Evaluation Limitations and Generalizability Constraints

The ten-participant sample, composed of six expert evaluators and four airport passengers, provides initial positive indicators for system acceptance but carries significant limitations:

- Ten participants is appropriate for exploratory pilot evaluation but insufficient for population-level conclusions regarding passenger acceptance.
- Six of ten participants were airport and project staff with domain expertise and prior system familiarity. This composition may have resulted in higher satisfaction ratings compared to the general public unfamiliar with emerging technologies.
- The four passenger evaluators were a convenience sample at a specific trial location during a specific time period. This does not represent international airport passenger diversity in terms of age, language, digital literacy, cultural background, or travel frequency.
- Participants evaluated the system in isolation rather than comparing against a baseline of traditional airport information channels.

Large-scale validation requires (1) substantially larger participant pools (200+ passengers) with demographic representation; (2) longitudinal studies tracking passenger behavior over weeks/months rather than single trial days; (3) pre/post deployment comparison with measurable operational metrics; and (4) evaluation across diverse airport terminal types and international locations.

### 5.11. Multilingual Support and International Passenger Accessibility

The humanoid robot’s user interface currently operates in English and Greek through its touchscreen display and text-to-speech functions. For Athens International Airport (a major European hub serving international passengers) this language limitation significantly restricts system usability for non-Greek-speaking passengers unfamiliar with English.

Future production deployments should prioritize multilingual support across at least english (international aviation standard), greek (local language), german (major EU passenger source), french (international aviation standard) and additional languages based on passenger origin analysis.

- The system architecture supports multilingual extension through API integration with cloud-based translation and text-to-speech services. Language detection can occur through voice recognition or manual touchscreen selection. Implementing multilingual support is technically feasible and should be prioritized for commercial deployments.

### 5.12. Robot as Information Intermediary: Comparative Value Analysis

- The humanoid robot’s role in the system requires nuanced understanding relative to alternative information delivery mechanisms. While information provision alone (flight status, gate assignments, wayfinding) could be delivered through alternative channels including dedicated mobile applications, airport Wi-Fi portals, or SMS notifications, the robot provides several distinctive advantages.
- Passengers without smartphones, airport network connectivity, or prior travel experience can access information through human–robot interaction.
- Dynamic question-answering and contextual assistance beyond pre-programmed information.
- Physical robot presence provides wayfinding guidance and passenger direction capabilities unavailable through digital channels.
- Real-time alerts based on thermal camera analytics enable dynamic routing recommendations.
- A well-designed mobile application could provide comparable informational content and potentially greater personalization for tech-savvy passengers connected to airport networks. However, such an application would not address passengers without smartphones, non-English/Greek speakers without access to airport-specific apps, or passengers preferring in-person interaction. The robot serves a complementary role within a multi-channel information strategy rather than functioning as the exclusive information delivery mechanism.
- Quantifying passenger throughput improvement attributable specifically to the humanoid robot is methodologically challenging without controlled experimental design comparing robot-assisted vs. non-assisted passenger flows. We recommend future research including:
- A/B testing scenarios with robot availability/unavailability.
- Measurement of corresponding passenger flow metrics (queue length, transit time, route selection).
- Comparative user experience between robot interface and alternative channels.
- Passenger preference studies across demographic groups.

## 6. Conclusions

This paper presented the implementation, deployment, and comprehensive evaluation of an AI-powered service robot ecosystem integrated with thermal camera networks and 5G connectivity for smart airport operations at Athens International Airport. The research successfully demonstrated the technical viability and practical applicability of advanced robotics systems for enhancing passenger experience while optimizing operational efficiency in high-traffic transportation environments.

### 6.1. Key Findings and Contributions

The UC11 trial achieved strong technical performance across all evaluated metrics, establishing new benchmarks for human–robot interaction systems in airport environments. The application round-trip latency of 42.9 milliseconds significantly outperformed the 800-millisecond requirement threshold, demonstrating the system’s capability to support real-time conversational interfaces essential for natural passenger assistance. Perfect service reliability and availability scores of 100% validated the robustness of the integrated 5G network infrastructure and AI-powered analytics platform under operational conditions.

The consistent positive user satisfaction ratings across all six Key Value Indicators (Trust (4.5), User Experience (4.3), Acceptability (4.3), Digital Inclusion (4.1), Resource Optimization (4.4), and Operational Efficiency (4.4)) represent a significant achievement in human–robot interaction research (see also Appendix A). These results demonstrate strong acceptance levels for AI-powered service robots among both passengers and airport personnel, indicating strong potential for widespread adoption in transportation infrastructure.

The privacy-by-design approach using thermal cameras successfully addressed GDPR compliance requirements while maintaining comprehensive passenger flow monitoring capabilities. This methodology provides a practical framework for balancing security needs with privacy protection in public spaces, addressing critical concerns for smart city applications. The system’s ability to generate real-time crowd analytics, congestion detection, and flow optimization without capturing personally identifiable information represents a significant contribution to privacy-compliant surveillance technologies.

### 6.2. Technical Achievements and System Performance

The integrated system architecture demonstrated seamless operation across multiple technology domains including thermal imaging, AI analytics, humanoid robotics, and 5G wireless networks. The wi.move platform successfully processed video streams from eight strategically deployed cameras while coordinating humanoid robot navigation, passenger assistance, and terminal operations supervision through a unified interface.

Network performance analysis revealed critical requirements for large-scale deployment, including uplink throughput of approximately 5 Mbps per robot for continuous video streaming and downlink requirements of 2 Mbps per robot for control signals. The round-trip latency averaging 20–25 milliseconds for MQTT traffic and 25–30 milliseconds during video streaming established baseline performance specifications for multi-robot environments, though occasional latency spikes above 40 milliseconds highlighted areas for improvement.

The zero packet loss achievement throughout all testing scenarios confirmed the stability and reliability of the 5G network infrastructure under operational airport conditions. This performance validation provides confidence for scaling the system to support multiple concurrent robots and expanded sensor networks across larger terminal areas.

### 6.3. Implications for Smart Airport Development

The trial results demonstrate the substantial potential for AI-powered robotics to transform airport operations through enhanced passenger assistance, automated flow management, and improved resource utilization. The system’s ability to reduce passenger wait times, accelerate service delivery, and provide actionable insights for Terminal Operations Supervisors validates the business case for smart airport investments.

Operational efficiency improvements including reduced staffing requirements and enhanced customer satisfaction translate to measurable return on investment for airport operators. The comprehensive data analytics capabilities enable predictive insights for capacity planning, security optimization, and passenger flow management, supporting strategic decision-making for airport infrastructure development.

The successful integration with existing airport systems including flight information displays, security protocols, and terminal operations demonstrates the system’s compatibility with legacy infrastructure. This interoperability reduces deployment complexity and supports incremental adoption strategies for widespread implementation.

### 6.4. Future Research Directions and 6G Requirements

The trial identified critical network performance requirements that inform 6G technology development for intelligent transportation systems. Ultra-low latency below 1 millisecond, deterministic communication with minimal jitter, and massive machine-type communications will enable more sophisticated multi-robot coordination and real-time collaborative operations.

Dynamic resource allocation and intelligent load balancing become essential for supporting hundreds of monitoring cameras and dozens of service robots required for comprehensive airport coverage. The integration of edge computing architectures, network slicing capabilities, and AI-as-a-Service functionality will provide the infrastructure foundation for next-generation smart airports.

Future research should focus on swarm robotics coordination algorithms, distributed AI processing architectures, and adaptive network resource management for large-scale deployments. Investigation of integration with digital twin technologies, predictive maintenance systems, and sustainable energy management represents critical areas for comprehensive smart airport ecosystems.

### 6.5. Limitations and Considerations

While the trial demonstrated successful proof-of-concept implementation, single-robot deployment scenarios do not fully address multi-robot coordination challenges, potential interference issues, or resource contention under peak traffic conditions. Extensive scalability testing remains necessary to determine system performance with multiple concurrent robots and hundreds of simultaneous users.

The jitter variation affecting system responsiveness highlights the need for more deterministic communication capabilities in future network deployments. Enhanced cybersecurity measures including quantum-resistant cryptography and blockchain-based authentication will become essential for large-scale airport implementations addressing evolving security threats.

Environmental impact assessment, energy efficiency optimization, and sustainable technology integration require systematic evaluation to ensure responsible innovation in aviation infrastructure development. The long-term operational costs, maintenance requirements, and staff training implications must be thoroughly analyzed before widespread commercial deployment.

### 6.6. Final Remarks

The UC11 smart airport ecosystem trial successfully validated the integration of AI-powered service robots, thermal camera networks, and 5G connectivity for enhancing passenger experience and optimizing airport operations. The strong technical performance metrics, consistent positive user satisfaction ratings, and successful real-world deployment under operational conditions establish a strong foundation for the future of intelligent transportation infrastructure.

The research contributions include novel privacy-compliant monitoring methodologies, comprehensive human–robot interaction evaluation frameworks, and practical 5G network performance specifications for large-scale robotics deployments. These findings provide valuable insights for airport operators, technology developers, and policymakers advancing smart city initiatives globally.

The alignment between objective technical measurements and subjective user perception confirms the system’s robustness, usability, and scalability potential for complex transportation environments. As airports worldwide face increasing passenger volumes and operational complexity, AI-powered robotics systems offer notable solutions for maintaining service quality while optimizing resource efficiency.

The successful UC11 implementation represents a significant step toward realizing the vision of fully autonomous, intelligent airport ecosystems that seamlessly integrate human needs with technological capabilities. Continued research, development, and strategic investment in these technologies will shape the future of aviation infrastructure and passenger experience for decades to come.

## Figures and Tables

**Table 1:** Operational Efficiency Metrics—Subjective Perception and Objective Measures.

Metric	Type	Value	Source
Operational Efficiency KVI Rating	Subjective (Likert)	4.4/5.0	Survey (*n* = 10)
Underlying Questions	Subjective	(1) System reduces wait times; (2) System provides actionable insights	Survey
Average Queue Length (Check-In)	Objective	8.2 persons (trial period)	Thermal cameras
Average Queue Length (Security)	Objective	6.4 persons (trial period)	Thermal cameras
Average Queue Length (Gate 1)	Objective	3.1 persons (trial period)	Thermal cameras
Passenger Flow Rate (average)	Objective	2.3 persons/min (trial location)	Thermal cameras
Peak Flow Rate	Objective	4.1 persons/min (mid-trial)	Thermal cameras

## Data Availability

Data is unavailable due to privacy restrictions.

## Abbreviations

The following abbreviations are used in this manuscript:

5G	Fifth-Generation Wireless Networks
6G	Sixth-Generation Wireless Networks
A/B	Comparison Testing (Variant A vs. Variant B)
AI	Artificial Intelligence
AIaaS	Artificial Intelligence as a Service
AIA	Athens International Airport
API	Application Programming Interface
B5G	Beyond Fifth-Generation Wireless Networks
CCTV	Closed-Circuit Television
DPA	Data Protection Authority
EDPB	European Data Protection Board
EET	Eastern European Time
Fps	Frames Per Second
GDPR	General Data Protection Regulation
HRLLC	Hyper-Reliable Low-Latency Communication
HTTP	Hypertext Transfer Protocol
IoT	Internet of Things
IoU	Intersection over Union
KMh	Kilometers Per Hour
KPI	Key Performance Indicator
KVI	Key Value Indicator
LIDAR	Light Detection and Ranging
LWIR	Long-Wave Infrared
M	Meter
mAP	Mean Average Precision
MHz	Megahertz
mK	Millikelvin
Mbps	Megabits Per Second
MQTT	Message Queuing Telemetry Transport
Ms	Millisecond
Μm	Micrometer
PII	Personally Identifiable Information
QMR	Quarterly Management Review
REST	Representational State Transfer
RGB	Red-Green-Blue (Color Space)
ROS 2	Robot Operating System 2
RTAB-Map	Real-Time Appearance-Based Mapping
SLAM	Simultaneous Localization and Mapping
TCP	Transmission Control Protocol
TLS	Transport Layer Security
TOS	Terminal Operations Supervisor
UC11	Use Case 11: Service Robots for Enhanced Passenger Experience
URLLC	Ultra-Reliable Low-Latency Communication
WebRTC	Web Real-Time Communication
wi.move	Integrated Smart Airport Management Platform (Project-specific system)
ZMQ	ZeroMQ (Message Queue Protocol)

## Appendix A

User Experience Questionnaire—Detailed Items

Administered to n = 10 trial participants using 5-point Likert scale (1 = Strongly Disagree, 5 = Strongly Agree)

TRUST Dimension (2 items)

1. I am confident that the system securely handles passenger data and flight information
2. I trust that system alerts about congestion are accurate and helpful

USER EXPERIENCE Dimension (2 items)

3. The robot’s touchscreen interface is intuitive and easy to navigate
4. The visual display of flight information and congestion maps is clear and understandable

ACCEPTABILITY Dimension (2 items)

5. The system is easy to use even during peak passenger traffic periods
6. I feel comfortable interacting with the robot for extended periods during high-traffic scenarios

DIGITAL INCLUSION Dimension (1 item)

7. The system is accessible to passengers with varying levels of digital literacy and device capabilities

RESOURCE OPTIMIZATION Dimension (2 items)

8. The system efficiently utilizes staff and robotic resources to streamline airport operations
9. The system reduces idle time for both robotic and human airport personnel

OPERATIONAL EFFICIENCY Dimension (1 item)

10. The system effectively reduces passenger wait times and accelerates airport operations

Mean scores by dimension:

- Trust: 4.5 ± 0.5
- User Experience: 4.3 ± 0.7
- Acceptability: 4.3 ± 0.6
- Digital Inclusion: 4.1 ± 0.8
- Resource Optimization: 4.4 ± 0.5
- Operational Efficiency: 4.4 ± 0.6

## Appendix B

The eight thermal cameras deployed across Athens International Airport utilize uncooled microbolometer sensor technology operating in the long-wave infrared (LWIR) spectral range from 8 to 14 micrometers, which is optimal for detecting human thermal signatures in crowded airport environments. Each camera captures video at a resolution of 640 × 480 pixels with a frame rate of 30 frames per second, providing sufficient spatial and temporal detail for accurate human detection and tracking. The cameras feature radiometric temperature measurement capabilities spanning a range from −20 °C to +120 °C with exceptional thermal sensitivity of 50 milliKelvin (mK), enabling detection of subtle temperature variations associated with human presence. The optical system employs a 24-degree horizontal field of view lens configuration that was strategically selected to provide comprehensive coverage of designated monitoring zones including check-in areas, security checkpoints, and terminal entrances while minimizing the number of required camera installations.

The system’s network architecture was designed to support real-time video streaming and bidirectional communication between distributed sensors, the humanoid robot, and the centralized wi.move platform. Each thermal camera generates a continuous video stream requiring approximately 2 to 3 megabits per second (Mbps) of uplink bandwidth when utilizing H.265 video compression encoding, which provides substantial bandwidth savings compared to H.264 encoding while maintaining detection accuracy. The humanoid robot’s telemetry data transmission via MQTT protocol consumes approximately 0.5 Mbps of bandwidth for continuous status updates, sensor readings, and location information. Robot uplink requirements, including onboard camera feeds and environmental sensor data, total approximately 5 Mbps per robot, while downlink bandwidth for control signals and command transmission averages 2 Mbps per robot. For the complete system configuration deployed during the UC11 trial—comprising eight thermal cameras and one humanoid robot—the aggregate network bandwidth requirement ranges from 22 to 25 Mbps, well within the capacity of modern 5G network infrastructure.

Comprehensive latency measurements were conducted throughout the trial period to evaluate the real-time responsiveness of the integrated smart airport ecosystem. Video frame processing latency from thermal camera capture through the wi.move AI detection pipeline averages 45 milliseconds per 640 × 480 pixel frame, including network transmission time, inference processing on edge GPU hardware, and result delivery to the dashboard interface. MQTT telemetry communication between the humanoid robot and the wi.move platform exhibits round-trip latency ranging from 20 to 25 milliseconds for low-data-volume messages such as position updates and status notifications. Video streaming communication via WebRTC protocol demonstrates round-trip latency between 25 and 30 milliseconds under normal network conditions, with occasional spikes to 40 milliseconds during periods of elevated network traffic. Robot command response time, measured from dashboard control input to robot actuation, averages 15 to 20 milliseconds, enabling near-instantaneous remote operation capabilities. Dashboard user interface updates reflecting new passenger flow statistics and congestion alerts occur within 50 to 70 milliseconds from the moment thermal camera data is processed, providing Terminal Operations Supervisors with timely situational awareness for decision-making.

The human detection and tracking system employs a customized YOLOv11-based deep convolutional neural network architecture specifically optimized for thermal imaging applications in crowded public spaces. The model was trained using a dataset comprising over 50,000 annotated thermal image frames collected at Athens International Airport under diverse operational conditions including varying passenger densities, lighting conditions, and environmental temperatures. The training dataset included examples of passengers in multiple poses and activities representative of typical airport scenarios, ensuring robust detection performance across realistic use cases. Model evaluation on an independent validation dataset demonstrates a mean average precision (mAP) of 0.87 at an Intersection over Union (IoU) threshold of 0.50, indicating strong detection accuracy for the intended application. Inference processing occurs on NVIDIA Jetson AGX edge GPU hardware co-located with the thermal cameras, achieving a detection latency of 45 milliseconds per frame, which enables real-time processing of all eight camera streams simultaneously without introducing significant delays in alert generation or dashboard updates.

The AI detection model’s performance was systematically evaluated across multiple human pose categories relevant to airport passenger monitoring applications. Standing passengers, who present the largest and most consistent thermal signatures, achieve the highest detection accuracy at 92 percent, with minimal false negatives even in moderately crowded scenes. Walking passengers maintain strong detection performance at 89 percent accuracy despite motion blur and variable thermal profiles associated with body movement and heat dissipation patterns. Sitting passengers present greater detection challenges due to reduced thermal signature visibility and partial occlusion by seating infrastructure, resulting in 74 percent detection accuracy, though this remains adequate for congestion monitoring purposes where seated individuals contribute less to active passenger flow dynamics. Passengers in bending or crouching positions achieve 81 percent detection accuracy, with performance variability dependent on the degree of body flexion and orientation relative to camera viewing angles. These pose-specific accuracy measurements demonstrate that while the system performs optimally for standing and walking passengers who dominate active flow patterns, it maintains acceptable detection capabilities across the full range of passenger behaviors encountered in operational airport environments.

Beyond individual person detection, the system implements a congestion classification algorithm that categorizes designated monitoring zones as either crowded or uncrowded based on configurable passenger density thresholds. This congestion classification function, which serves as the system’s primary operational capability for triggering Terminal Operations Supervisor alerts and passenger routing recommendations, achieves an overall accuracy of 94 percent when evaluated against ground-truth annotations of crowded versus uncrowded conditions. The classification algorithm demonstrates high sensitivity with a true positive rate of 96 percent, meaning it reliably detects genuine congestion situations with minimal missed alerts that could compromise operational awareness. Specificity, measured as the true negative rate, reaches 92 percent, indicating the system effectively avoids false congestion alerts that could undermine operator confidence or trigger unnecessary interventions. The congestion threshold employed during the trial was configured at 15 or more persons present within a defined region of interest, though this threshold can be dynamically adjusted based on specific operational requirements, terminal geometry, and passenger flow patterns. This congestion-focused operational paradigm acknowledges that absolute passenger counting accuracy, while valuable, is less critical than reliable detection of threshold-exceeding crowd conditions that require operational attention or passenger routing interventions.

The humanoid service robot operates using Robot Operating System 2 (ROS 2) with the Nav2 navigation stack for dynamic path planning and obstacle avoidance in real-time. The robot employs RTAB-Map (Real-Time Appearance-Based Mapping) SLAM algorithm for simultaneous localization and mapping, enabling it to build and maintain a three-dimensional representation of the airport terminal environment while continuously tracking its position with centimeter-level accuracy. Onboard sensors include a forward-facing RGB camera for visual odometry and landmark recognition, a 360-degree scanning LIDAR sensor providing distance measurements up to 30 m with angular resolution of 0.25 degrees, and an inertial measurement unit (IMU) comprising accelerometers and gyroscopes for motion tracking and orientation estimation. The robot’s autonomous navigation system supports predefined waypoint sequences that can be dynamically modified based on real-time passenger density information received from the thermal camera network, enabling intelligent positioning in high-traffic areas where passenger assistance demand is greatest. Maximum autonomous navigation speed is configured at 0.8 m per second in crowded environments and 1.2 m per second in open spaces, with dynamic speed adjustment based on proximity to detected obstacles and pedestrian traffic patterns.

The system implements multiple communication protocols optimized for different data types and quality-of-service requirements. Thermal camera video streams utilize WebRTC (Web Real-Time Communication) protocol, which provides encrypted peer-to-peer data transmission with ultra-low latency characteristics essential for real-time video analytics applications. Robot telemetry and control messaging employ MQTT (Message Queuing Telemetry Transport), a lightweight publish-subscribe protocol designed for constrained devices and networks, enabling efficient bidirectional communication between the robot and the wi.move platform with minimal overhead. Bulk data transfers, including historical video archives and large configuration files, utilize ZMQ (ZeroMQ) over TCP (Transmission Control Protocol), which provides reliable delivery with flow control and error recovery mechanisms. All communication channels implement Transport Layer Security (TLS) encryption to protect data confidentiality and integrity during transmission over the 5G network infrastructure. The wi.move platform exposes RESTful HTTP APIs (Representational State Transfer over Hypertext Transfer Protocol) for third-party integration, enabling Terminal Operations Supervisors to access passenger flow statistics, retrieve historical analytics, and configure system parameters through standardized web service interfaces compatible with existing airport management information systems.

The wi.move platform implements a dual-database architecture optimized for different query patterns and data retention requirements. Time-series passenger flow metrics including per-camera counts, queue lengths, flow rates, and congestion indicators are stored in InfluxDB, a specialized time-series database that provides efficient storage and rapid retrieval of timestamped measurements. InfluxDB query performance for real-time dashboard updates averages 5 to 15 milliseconds for queries spanning the most recent 60 min of data, ensuring responsive visualization of current operational status. Historical aggregated statistics, including daily passenger throughput summaries, hourly congestion pattern analyses, and long-term trend data, are maintained in PostgreSQL, a mature relational database management system offering robust transaction support, complex query capabilities, and data integrity guarantees. Video retention policies implement automatic deletion of raw thermal camera feeds after 72 h while preserving derived statistics and alert records indefinitely for operational analysis and system performance evaluation. This tiered storage approach balances real-time operational requirements with long-term analytics capabilities while managing storage costs and complying with data minimization principles required under GDPR.

The distributed computing architecture comprises edge processing nodes co-located with thermal camera clusters and cloud-based centralized services hosted on the wi.move platform infrastructure. Each edge processing node incorporates an NVIDIA Jetson AGX Xavier embedded GPU platform featuring 512-core Volta GPU architecture with 64 Tensor Cores, providing 32 teraflops of AI inference performance at 30-watt power consumption suitable for deployment in terminal infrastructure cabinets. The Jetson platform runs containerized inference services using Docker, enabling rapid deployment, version management, and horizontal scaling as additional camera zones are added. Centralized cloud services operate on a Kubernetes cluster with auto-scaling capabilities, ensuring sufficient computational resources are available during peak operational periods while minimizing idle infrastructure costs during low-traffic hours. The hybrid edge-cloud architecture enables latency-sensitive functions such as real-time human detection, tracking, and local alert generation to execute at the edge with sub-50-millisecond response times, while computationally intensive batch analytics, historical trend analysis, and machine learning model training occur in the cloud with access to greater computational and storage resources.
